# Analgesic-Like Activity of Essential Oil Constituents: An Update

**DOI:** 10.3390/ijms18122392

**Published:** 2017-12-09

**Authors:** Rita de Cássia da Silveira e Sá, Tamires Cardoso Lima, Flávio Rogério da Nóbrega, Anna Emmanuela Medeiros de Brito, Damião Pergentino de Sousa

**Affiliations:** 1 Departamento de Fisiologia e Patologia, Universidade Federal da Paraíba, CP 5009, João Pessoa-PB 58051-970, Brazil; ritacassia.sa@bol.com.br; 2 Departamento de Farmácia, Universidade Federal de Sergipe, São Cristóvão-SE 49100-000, Brazil; tamires.cl87@gmail.com; 3 Departamento de Ciências Farmacêuticas, Universidade Federal da Paraíba, João Pessoa-PB 58051-970, Brazil; frnobrega@hotmail.com (F.R.N.); manubbrito@hotmail.com (A.E.M.B.)

**Keywords:** analgesic, antinociceptive, terpenes, phenylpropanoid, natural products, medicinal plants, aromas, flavor, volatile, food

## Abstract

The constituents of essential oils are widely found in foods and aromatic plants giving characteristic odor and flavor. However, pharmacological studies evidence its therapeutic potential for the treatment of several diseases and promising use as compounds with analgesic-like action. Considering that pain affects a significant part of the world population and the need for the development of new analgesics, this review reports on the current studies of essential oils’ chemical constituents with analgesic-like activity, including a description of their mechanisms of action and chemical aspects.

## 1. Introduction

Constituents of essential oils are commonly found in foods giving characteristic aroma and flavor. Several plants and their fruits can be recognized by the aroma provided by volatile substances, which are generally alcohols, aldehyde ketones, esters, hydrocarbons, phenols, among other chemical classes. For example, alcohol linalool can be found in mango, papaya and pineapple fruit. While the hydrocarbon limonene is present in orange, lemon and guava [1]. In cooking, the aroma of spices is due to the presence of these volatile compounds, such as phenol eugenol found in clove (*Syzygium aromaticum* (L.) Merrill & Perry) [2]. Therefore, people are always in contact with these constituents through food.

Essential oils are a class of natural products with promising biological properties and are traditionally used in aromatherapy for various purposes. Pharmacological and clinical studies have demonstrated the profile of these compounds as drug candidates [3]. For example, the monoterpene perillyl alcohol has preventive and therapeutic effects in a wide variety of preclinical tumor models and is currently under phase I and phase II clinical trials, including against glioblastomas multiforme. Elemene and d-limonene are essential oil constituents also tested in patients with cancer [3]. In fact, several reviews have suggested the therapeutic potential of this group into multiple areas, including analgesics, whose activity presents a large number of published studies [4,5], anticonvulsants [6], anti-inflammatories [7,8,9], anticancer agents [10,11], anxiolytics [12], and antiulcer agents [13]. Studies of the anxiolytic effects have scientifically proven the therapeutic use of several essential oils in aromatherapy. The inhalation route is an interesting route for several therapeutic approaches using these natural products [3,12]. The chemical diversity found in essential oils may be responsible for these variety of pharmacological activities and possibly the various mechanisms of action of their chemical constituents. These findings not only support the traditional use of aromatic plants and their essential oils but also highlight analgesic-like uses of these natural products. In addition, antitumor essential oils with pharmacological activity in animal models of pain may have a dual effect on the therapeutic approach of patients with cancer. Pain is defined as an unpleasant sensory and emotional experience associated with actual or potential tissue damage, or described in terms of such damage [14]. This symptom affects a lot of people in the world, mainly patients with some types of chronic pathologies, causing loss of good quality of life. In response to the demand for powerful analgesics and with less side effects many studies have been conducted to discover bioactive substances with profiles of analgesic drug candidates. Therefore, with the aim of contributing to the report of natural compounds with antinociceptive activity, the purpose of this review was to conduct a systematic investigation of studies on the essential oil constituents in experimental models related to antinociceptive activity. This article is an update of our latest review on the topic [4] and describes new studies conducted over the past six years.

## 2. Results

### 2.1. p-Cymene

The aromatic monocyclic monoterepene *p*-cymene [1-methyl-4-(1-methylethyl) benzene] is the biological precursor of carvacrol and is abundantly found in essential oils from many plant species such as *Protium heptaphyllum* (Aubl.) Marchand (Burseraceae) [15] and *Hyptis pectinata* (L.) Poit. (Lamiaceae) [16]. It also occurs naturally in a wide variety of foods, including orange juice, carrots, tangerine, butter and oregano [17]. The antinociceptive and anti-inflammatory activities of *p*-cymene have been evaluated by different behavioral tests of nociception in rodents, which showed that this monoterpene exerted both peripheral and central antinociceptive action. For instance, the antinociceptive effect of *p*-cymene was demonstrated on an orofacial nociceptive response model through tests involving the subcutaneous administration of formalin, capsaicin, and glutamate into the upper lip of *p*-cymene-pretreated male Swiss mice (25, 50 or 100 mg/kg, i.p.). *p*-Cymene markedly decreased the rubbing behavior induced by all three components; an effect counteracted by nonselective opioid receptor antagonist naloxone, indicating the participation of the opioid system in the antinociceptive response [18].

A study developed by Bonjardim and collaborators [19] revealed that, in the acetic acid-induced writhing and formalin tests, exposure of male Swiss mice to *p*-cymene (50 or 100 mg/kg, i.p.) significantly decreased the number of writhes and the licking time in the first and second phase of the formalin test. In the hot plate test, it increased the latency time of the licking and jumping behavior to thermal stimulus. Additionally, *p*-cymene (25, 50 or 100 mg/kg) produced an anti-inflammatory reaction induced by carrageenan (CG), which led to a marked reduction in leukocyte migration. In mice, intraplantar injection of CG leads to hypernociception and an inflammatory response that involves the release of cytokines by resident or migrating cells initiated by the production of bradykinin [20]. This is followed by the secretion of prostanoids and sympathomimetic amines, such as dopamine [21], which stimulate Aδ and C fiber nerve terminals and the release of substance P and neurokinin A, accentuating local blood flow and vascular permeability [22]. In CG-induced hypernociception, the release of tumor necrosis fator α (TNF-α) and keratinocyte-derived chemokine (KC), for exemple, is accompanied by the secretion of interleukin 1β (IL-1β) [23] with the subsequent induction of cyclooxigenase-2 (COX-2) expression and the production of protanoids, such as prostagladin E2 (PGE2) [24]. 

Other studies have provided further evidence of the antinociceptive and anti-inflammatory properties of *p*-cymene and the possible role of the opioid system and cytokines in these responses. The antinociceptive effect of *p*-cymene (25–100 mg/kg) on male Swiss mice was demonstrated in the tail flick test, showing increased dose-dependent reaction time, an effect that lasted for five hours and was antagonized by naloxone and by δ, κ and µ-opioid receptor antagonists naltrindole, nor-binaltorphimine (Nor-BNI) and CTOP (D-Phe-Cys-Tyr-D-Trp-Orn-Thr-Pen-Thr amide), respectively [25]. In the assessment of the anti-inflammatory activity, treatment with *p*-cymene (25, 50 or 100 mg/kg, i.p.) decreased mechanical hyperalgesia induced by CG, TNF-α, PGE2, and dopamine. In the CG-induced pleurisy test, *p*-cymene reduced leukocyte (100 mg/kg) and neutrophils (50 and 100 mg/kg) migration to the pleural cavity, and decreased the levels of TNF-α in pleural exudates (25, 50 and 100 mg/kg). Neutrophils, in particular, play an important role at the onset of inflammatory hypernociception by secreting pro-inflammatory cytokines (e.g., TNF-α and IL-1β) and mediators such as prostaglandins [26,27]. *p*-Cymene was also shown to diminish nitric oxide (NO) production in murine macrohages incubated with lipopolysaccharide (LPS) (25, 50 and 100 µg/mL), a component known to stimulate toll-like receptor 4 (TLR-4), leading to the activation of the transcription factor NF-κB [25]. NF-κB contributes to the production of inflammatory (pro-nociceptive) molecules and enhances inducible nitric oxide synthase (iNOS) activity, thereby increasing NO production [28], which is believed to act as a mediator of inflammation and to sustain hyperalgesia after CG injection [29]. Furthermore, *p*-cymene significantly enhanced the c-Fos immunoreactive neurons in the periaqueductal gray [25], a midbrain region activated by opioid agonists and involved with pain modulation [30]. Together these findings indicate an anti-inflammatory and antinociceptive action of *p*-cymene and suggest the involvement of descending pain suppression mechanisms since its antinociceptive role through the opioid system was increased by the activation of the periaqueductal gray [25].

The assessment of the antinociceptive property and redox profile of *p*-cymene and two other monoterpenes, namely (+)-camphene and geranyl acetate, revealed that *p*-cymene possessed the strongest antinociceptive action (50, 100 and 200 mg/kg, i.p.) while (+)-camphene and geranyl acetate (200 mg/kg) displayed a moderate analgesic effect in male Swiss mice tested in the acetic acid-induced writhing and formalin models [31]. In contrast, (+)-camphene exhibited the most relevant antioxidant effect in vitro detected by two specific assays: the thiobarbituric acid-reactive species (TBARS)—an assay employed to quantify lipid peroxidation [32]—and the total reactive antioxidant potential (TRAP)/total antioxidante reactivity (TAR)—an assay employed to estimate the nonenzymatic antioxidant capacity of samples [33]. It also showed the highest scavenging activity against different free radicals, including hydroxyl and superoxide radicals [31]. 

### 2.2. Carvacrol

Carvacrol (5-isopropyl-2-methylphenol) is a phenolic monoterpene found in essential oils of plants from the genera *Origanum* and *Thymus* (Lamiaceae) [34,35]. Its phamacological properties include acetylcholinesterase inhibition [36], and anticonvulsive [37], anxiolytic [38], and antinociceptive [39] action. The antinociceptive activity of carvacrol was demonstrated in male Swiss mice tested in animal models of pain (acetic acid-induced writhing, formalin and hot plate). The data obtained after oral treatment with single doses of carvacrol showed a decrease in the number of constrictions (50, 100 and 200 mg/kg), and the paw-licking time (50 mg/kg, first phase of the formalin test; 100 mg/kg, first and second phases), and an increase in the reaction time at 60 min (50 and 100 mg/kg) in the hot plate test. These effects were not reversed by naloxone and l-arginine, suggesting that the antinociceptive action of carvacrol may not be related to the opioid system [40]. On the other hand, the antinociceptive activity of carvacrol was associated with the inhibition of prostaglandin synthesis [39] as it possesses an effective ability to suppress COX-2 expression and to activate the peroxisome proliferator-activated receptors (PPAR) α and γ [41]. 

In a study by Guimarães and collaborators [42], the role of carvacrol in the attenuation of mechanical hypernociception and inflammation was investigated in models of hypernociception induced by CG, TNF-α, PGE2 and dopamine, and in models of CG-induced pleurisy, paw edema, and LPS-induced nitrite production in murine macrophages. The administration of carvacrol (50 or 100 mg/kg, i.p.) to male Swiss mice significantly suppressed mechanical hypernociception and paw edema induced by CG and TNF-α (but not PGE2 and dopamine), and markedly reduced TNF-α levels in pleural lavage, blocked leukocytes recruitment, and decreased LPS-induced nitrite production in vitro (carvacrol: 1, 10 or 100 µg/mL). Additionally, Guimarães and collaborators [43] also demonstrated the antinociceptive effect of carvacrol in the formalin-, capsaicin-, and glutamate-induced orofacial nociception tests in which male Swiss mice exhibited reduced face-rubbing behavior in both phases of the formalin test, and nociception induced by capsaicin and glutamate (carvacrol-25, 50 or 100 mg/kg, i.p.).

The antinociceptive action of carvacrol was further corroborated by a study developed by Luo and colloborators [44] in the assessment of its activity on glutamatergic spontaneous excitatory transmission in substantia gelatinosa neurons of the spinal dorsal horn, a region believed to modulate nociceptive transmission from the peripheral to the central nervous system [44,45]. By the use of the patch-clamp method in adult rat spinal cord slices, it was verified that exposure to carvacrol increased the secretion of l-glutamate from nerve terminals by activating transient receptor potential cation channels, subfamily A, member 1 (TRPA1), and produced membrane hyperpolarization; an effect that could be contributing to its anti-inflammatory action. Several studies have recognized TRP as important analgesic targets in inflammatory and neurophatic pains [46]. Another contribution was given by Joca and collaborators [47] that examined possible mechanisms involved in the effects of carvacrol on the peripheral nervous system. Carvacrol reversibly and dose-dependently suppressed the excitability of the rat sciatic nerve (IC_50_ value of 0.50 ± 0.04 mM), and prevented the generation of action potentials (IC_50_ 0.36 ± 0.14 mM) of the intact dorsal root ganglion (DRG) neurons without altering the resting potential and input resistance. Carvacrol also suppressed neuronal excitability by a direct inhibition of the voltage gated sodium current of dissociated DRG neurons (IC_50_ 0.37 ± 0.05 mM), suggesting a local anesthetic effect of this compound.

### 2.3. Linalool

(−)-Linalool is an enantiomer monoterpene present in essential oils of various aromatic plants, such as lavender, rosewood and bergamot [48], and possesses several pharmacological activities including anti-inflammatory, anxiolytic, anticonvulsant and antinociceptive [49,50,51,52,53]. The effects of (−)-linalool, extracted from the essential oil of *Ocimum basilicum* L. (Lamiaceae) leaf, on orofacial nociception were addressed in formalin, glutamate and capsaicin tests and in an electrophysiological protocol, which involved the evaluation of the neuronal excitability of the hippocampal dentate gyrus. (−)-Linalool (50, 100 and 200 mg/kg, i.p.) administered to male Swiss mice effectively inhibited the nocifensive face-rubbing behavior in the first and second phase of the formalin test. At high doses, it also reduced nociceptive behavior in neurogenic inflammatory nociception induced by capsaicin and glutamate injection in the perinasal area (right upper lip) [53]. It is believed that these effects are related to possible inhibition of substance P release or blocking effect on its receptor neurokinin-1 (NK-1) [54]. In addition, the electrophysiological analysis revealed that (−)-linalool inhibited the field potentials activated by the antidromic stimulation of the hylus, suggesting that this compound affects the activation of the voltage-dependent sodium channels present in the granular neurons of the hippocampal dentate gyrus [53,55]. Similar results were observed with the *O. basilicum* leaf essential oil, indicating that both the oil and (−)-linalool display modulatory action on neurogenic and inflammatory pain, and that the antinociceptive effect could be related to reduced peripheral and central nerve excitability [53].

The antinociceptive activity of (±)-linalool was evidenced in the paclitaxel-induced acute pain model in male ddY-strain mice. Intraplantar injection of (±)-linalool (5 and 10 µg/paw) effectively and dose-dependently suppressed behavioral responses of paclitaxel-induced mechanical allodynia and hyperalgesia. (±)-Linalool injected into the ipsilateral paw produced antiallodynia and antihyperalgesia effects whereas no such action was detected in the linalool-injected contralateral paw, suggesting that the effects exerted by this monoterpene may be mediated locally rather than systemically. Moreover, (±)-linalool’s effects were reversed by local (paw plantar surface) administration of naloxone hydrochloride (opioid antagonist) and by naloxone methiodide (peripherally acting opioid receptor antagonist), indicating that (±)-linalool’s peripheral antiallodynia and antihyperalgesia activities could partly involve peripheral opiod mechanisms [48].

Bergamot essential oil extracted from *Citrus bergamia* (Risso, Rutaceae) is a rich source of linalool. The investigation of their effects on neurophatic hypersensitivity induced by partial sciatic nerve ligation (PSNL) in male ddY-strain mice showed that intraplantar injection of these components into the ipsilateral hindpaw decreased PSNL-induced mechanical allodynia dose-dependently whereas no antinociceptive activity was observed after intraplantar injection into the contralateral hindpaw, further suggesting a local effect of linalool and also of bergamot essential oil [56]. The possible involvement of spinal extracellular signal-regulated protein kinase (ERK) in bergamot essential oil and linalool-induced antimechanical nociception indicates that the attenuation of the observed effects entailed inhibition of spinal ERK phosphorylation since intraplantar injection of bergamot essential oil or linalool effectively blocked spinal ERK activation induced by PSNL [56]. The activation of ERK has been demonstrated in dorsal horn neurons in persistent CG and Freund’s adjuvant-induced inflammatory hyperalgesia [57,58]. Previous studies have shown that injection of capsaicin into the hindpaw produced ERK activation in the spinal cord, while blockade of spinal ERK1/2 activity via i.t. injection of MEK inhibitor U0126 decreased nocifensive responses induced by formalin, capsaicin, CG or complete Freund’s adjuvant [59,60,61].

Corroboration of the local action of bergamot essential oil and linalool was provided by Katsuyama and collaborators [62]. The nocifensive response to formalin (licking and biting) was considerably decreased in both phases of the formalin test following intraplantar administration of bergamot essential oil or linalool into the ipsilateral, but not the contralateral, hindpaw of male ddY-strain mice. These findings show the peripheral antinociceptive action of both compounds, which was antagonized by intraplantar and i.p. injection of naloxone hydrochloride and naloxone methiodide, and confirm previous reports that suggest the involvement of peripheral opioid receptors in antinociception induced by bergamot essential oil and linalool [62]. 

In traditional Chinese medicine, frankincense from *Boswellia carterii* is commonly used for topical treatment of pain and inflammation [63]. A study carried out to investigate the antinociceptive and anti-inflammatory action of frankincense oil and water extracts and three of its main componentes, i.e., linalool, α-pinene and 1-octanol, via xylene-induced ear edema and a formalin-inflamed hindpaw model in male Kunming mice, showed consistent evidence about their anti-inflammatory and analgesic effects. Frankincense oil extract, which contains more linalool, α-pinene and 1-octanol than frankincense water extract, produced a faster and more effective reduction of the swelling and pain than the water extract. In addition, the combination of linalool, α-pinene and 1-octanol exhibited stronger biological effect on hindpaw inflammation and COX-2 overexpression than the three compounds used separately, indicating that they contribute to the topical antinociceptive and anti-inflammatory properties of frankincense by inhibiting COX-2 activation [64].

A study by Tashiro and collaborators [65] reported the antinociceptive effect of linalool in a different experimental protocol using vapour exposure mediated by hypothalamic orexin neurons, one of the main mediators in the behavioral responses to pain [66]. The involvement of these cells was evidenced by a significant increase in the number of c-Fos-expressing orexin neurons, and in linalool odour-exposed and odourless air-exposed orexin neuron-ablated mice that exhibited similar pain behavior in the first and second phase of the formalin test. The confirmation of the contribution of orexinergic transmission was shown in orexin peptide-deficient mice exposed to linalool vapour in which linalool failed to evoke antinociceptive effects after formalin-induced insult, suggesting the participation of orexinergic transmission in linalool odour-induced antinociceptive response. Moreover, linalool odour exposure significantly decreased pain response in both phases of the formalin test in mice (wild type: C57BL16) while, in the hot plate test, it increased the latency of hindpaw withdrawal when compared with the odourless air control following an injurious heat stimulus. In the investigation of the participation of olfactory processing in linalool analgesic effects by chemical nociceptive stimulus (formalin test), pain behavior in olfactory bulbectomized mice under linalool vapour exposure did not differ markedly from the odourless air group in both phases of the test. In the anosmic model using mice with a nonfunctional olfactory epithelium, no effects of linalool vapour were observed, providing further evidence that the olfactory response produced by linalool vapour may play a key role in inducing analgesic effects [65]. 

Despite the biological properties of (−)-linalool, its use in the treatment of painful and inflammatory disorders is still limited due to poor oral availability [67,68]. A comparative study using experimental pain models (i.e., acetic acid-induced writhing, formalin and hot plate) in male Swiss mice examined the antinociceptive effect of (−)-linalool and β-cyclodextrin (β-CD) complexed (−)-linalool (20 or 40 mg/kg, p.o.). Both compounds effectively reduced the nocifensive response in all chemical and heat-induced tests, suggesting the involvement of peripheral and central antinociceptive mechanisms. In the writhing test, the antinociceptive effects were antagonized by naloxone, implying the involvement of the opioidergic neurotransmission pathway. (−)-Linalool and (−)-linalool/β-CD complex also inhibited total leukocyte migration and TNF-α levels in peritoneal fluid in the CG-induced peritonitis protocol. However, (−)-linalool/β-CD complex exhibited stronger antinocicptive effect than (−)-linalool alone, indicating once again that cyclodextrin may become a relevant tool to improve the biological activity of water-insoluble monoterpenes [67].

Furthermore, the antinociceptive effect of (−)-linalool and β-CD complexed (−)-linalool was demonstrated in an animal model of chronic noninflammatory muscle pain (fibromyalgia animal model) [69], corroborating the findings by Quintans-Júnior and collaborators [67]. After exposure of male Swiss mice to (−)-linalool and (−)-linalool/β-CD complex (25 mg/kg, p.o.), the animals were tested for mechanical hyperalgesia (von Frey), motor coordination (rotarod), and muscle strength (grip strength meter) for 27 days. Both compounds markedly suppressed mechanical hyperalgesia in the model for fibromyalgia, persisting for 24 h only in the linalool complexed in β-cyclodextrin group. Additionally, the assessment of the areas in the central nervous system involved in antihyperalgesic activity by a method for immunofluorescence labeling of fos protein showed that both compounds effectively activated neurons of the locus coeruleus, nucleus raphe magnus and periaqueductal gray areas, suggesting the participation of descending pain pathways in the improved antinociceptive effect of (−)-linalool/β-CD complex [69].

### 2.4. Eugenol

Eugenol (2-methoxy-4-(2-propenyl) phenol) is a phenylpropanoid found as the main constituent of *Eugenia aromatica* (L.) Baill (clove oil, Myrtaceae) [70,71], being commonly used as an analgesic and anti-inflammatory in dental procedures, e.g., pulpitis and dentinal hypersensitivity [71,72,73]. Other phamacological properties of this compound include neuroprotective [74], anticonvulsant [75], antipyretic [76], and reduction of neuropathic [77] and orofacial pain [78]. The administration of eugenol (1–10 mg/kg, p.o.) has been reported to produced dose-dependent antinociceptive effects in male ICR mice (a strain of albino mice originated from the Institute of Cancer Research in the United States) tested in the acetic acid-induced writhing test—an effect that lasted for at least 30 min—and to inhibit the nociceptive behavior in the second phase of the formalin test as well as the nocifensive response time (reduced licking, scratching and biting of the lumbar or caudal region) for intrathecal injection of substance P (a neuropeptide associated with inflammatory processes and pain) or glutamate. Intraperitoneal pretreatment with naloxone and yohimbine (α2-adrenergic receptor antagonist) antagonized the analgesic effect of eugenol in the writhing test, whereas no such action was observed after pretreatment with methysergide (5-hydroxytryptamine (5-HT) serotonergic receptor antagonist) [73]. Bó and collaborators [71] provided more information about the mechanisms involved in the effect of eugenol on acute pain by indicating the participation of glutamatergic and TNF-α pathways. In the acetic acid-writhing test, exposure to eugenol (3–300 mg/kg, p.o., 60 min or i.p., 30 min) suppressed 82 ± 10% and 90 ± 6% of the nociceptive response of male Swiss mice (ID_50_ values of 51.3 and 50.2 mg/kg, respectively) while, in the glutamate test, eugenol (0.3–100 mg/kg, i.p.) decreased the response behavior by 62 ± 5% (ID_50_ of 5.6 mg/kg)—An effect that was reversed by naloxone. The administration of eugenol (10 mg/kg, i.p.) inhibited the nociception induced by the intrathecal (i.t.) injection of glutamate (37 ± 9%), kainic acid (kainite) (41 ± 12%), α-amino-3-hydroxy-5-methyl-4-isoxazolepropionic acid (AMPA) (55 ± 5%), substance P (SP) (39 ± 8%), and biting behavior induced by TNF-α (65 ± 8%). These findings give further evidence for the involvement of the opioid receptors in the antinociceptive action of eugenol and suggest that the mechanism of action also seems to include the modulation of glutamatergic receptors (i.e., kainate and AMPA), and the inhibition of TNF-α.

Eugenol is also the major constituent of the essential oil of *Ocimum gratissimum* L. (Lamiaceae), a plant popularly used in the treatment of painful diseases. In a model of neuropathic pain induced by chronic constriction of the sciatic nerve, the oral exposure to eugenol and the monoterpene myrcene (5 or 10 mg/kg, for 14 days after surgery) produced antihypernociceptive effects on male C57BL/6J mice tested in mechanical (von Frey) and thermal (hot plate) tests [79]. In addition, the administration of eugenol (1, 5 and 10 mg/kg) markedly decreased IL-1β levels whereas no significant effect was caused by treatment with myrcene. Similar antihypernociceptive activity was obtained with *O. gratissimum* essential oil (20 and 40 mg/kg) in the neurophatic pain models, providing evidence for the biological action that supports its popular use [79].

The evaluation of the antinociceptive effect of eugenol in a monoiodoacetate-induced osteoarthritis model revealed that daily administration of eugenol (40 mg/kg, p.o.) to Sprague Dawley rats during four weeks significantly changed gait parameters (e.g., swing speed, swing phase duration and duty cycle) of the treated hindlimb and reduced secondary mechanical allodynia in the first and the third week of treatment (measurement of withdrawal threshold in response to von Frey filaments). Spinal pain-related peptide analysis showed reduced expression of substance P and calcitonin gene-related peptide (CGRP) and increased levels of dynorphin (an opioid peptide) in animals exposed to eugenol [80]. It is known that the pharmacological inhibition of Transient Receptor Potential Vanilloid 1 (TRPV1) lowers the secretion of substance P [81] and as eugenol acts in the central nervous system, this may account for the decrease in substance P content [77]. Furthermore, the decrease in CGRP could be related to reduced activity of the rat knee joint afferent fibres since, like substance P, CGRP is also present in these cells [80,82]. These results indicate an effective antinociceptive action of eugenol to attenuate osteoarthritis-related pain [80].

In an experimental procedure using a half-tongue model in humans, Klein and collaborators [83] demonstrated that eugenol and carvacrol induced temporally desensitinzing patterns of oral irritation and increased innocuous warmth and noxious heat sensation on the tongue. The irritant sensation caused by both compounds was reduced during repeated applications at a 1 min interstimulus interval (self-desensitization) which lasted for at least 10 min. Cross-desensitization of capsaicin-evoked irritation was also observed. Eugenol and carvacrol elicited a significant increase in the magnitude of perceived innocuous warmth for at least 10 min, and briefly (less than 5 min) intensified heat pain at a 49 °C stimulus. It was suggested that the short-lived hyperalgesia after eugenol exposure may be associated with TRPV3-mediated improvement of thermal gating of TRPV1 present in lingual polymodal nociceptors [83]. 

Xixin (Asari Radix et Rhizoma) is a traditional herbal medicine used in China, Japan, and Korea as a local anesthetic, in inflammatory diseases, and to releave toothache and headache [84]. Methyl eugenol (4-allyl-1,2-dimethoxybenzene), a structural analogue of eugenol, is the main active component isolated from Xixin, known to possess antinociceptive, anesthetic, anticonvulsant, hypothermic and myorelaxant properties [75,85,86]. An in vitro study using cDNA clone of pNaEx8 plasmid encoding the Nav1.7 α subunit transiently expressed in Chinese hamster ovary (CHO) cells showed the inhibitory effect of methyl eugenol on Nav1.7 channels as a mechanism involved in its antinociceptive and anesthetic actions [86]. Methyl eugenol tonically suppressed peripheral nerve Nav1.7 currents in a dose- and voltage-dependent manner in the whole-cell patch-clamp method (IC_50_ of 295 μmoL/L at a −100 mV holding potential). Functionally, methyl eugenol expressed higher affinity to Nav1.7 channels in the inactivated and/or open state, indicating that, when in the presence of methyl eugenol, Nav1.7 channels presented decreased availability for activation in a steady-state inactivation protocol, strong use-dependent inhibition, increased binding kinetics, and slow recovery from inactivation compared to untreated channels. This suggests that the antinociceptive and anesthetic properties of methyl eugenol may be a consequence of the inhibitory effect of this compound on peripheral sodium channels [86]. 

In addition to methyl eugenol, another compound known as ortho-eugenol, a synthetic isomer of eugenol, has also been reported to cause antinociceptive and anti-inflammatory effects. In a study by Fonsêca and collaborators [87], the administration of ortho-eugenol (50, 75 and 100 mg/kg, i.p.) to male Swiss mice tested in the acetic acid writhing and glutamate tests caused a significant reduction in the number of writhes and in the licking time, respectively. The animals presented increased reaction time from thermal stimulus in the hot plate test, but treatment with yohimbine antogonized the antinociceptive activity, indicating a possible mechanism of action involving the adrenergic system. The anti-inflammatory action of ortho-eugenol was evidenced in inflammatory protocols, including the acetic acid-induced peritoneal permeability and the CG-induced peritonitis, which showed a suppressive effect on vascular permeability and leukocyte migration and the subsequent reduction of TNF-α and IL-1β due to inhibition of NF-κB and p38 phosphorilated forms observed in the peritonitis test.

### 2.5. Menthol

Menthol and l-menthol, predominant analgesic menthol isomer in medicinal preparations [88] is a natural cooling compound in peppermint oil from mint plants, and is found in various commercial products, e.g., toothpast, chewing gum and topical analgesics [89]. Low to moderate concentrations of topically applied menthol has been shown to inhibit capsaicin irritancy, sprains, heat hypersensitivity and headaches [90,91,92,93,94], while high concentrations (topical use or intraplantar injection) generated cold allodynia and hyperalgesia [94,95,96,97]. In addition, patients with neuropathic pain have been reported to also exhibited increased analgesic response induced by menthol [96].

The central mechanisms of analgesia induced by menthol was investigated by Pan and collaborators [94] in an in vitro assay using primary cultures of spinal cord superficial dorsal horn neurons obtained from 2- to 4-day-old CD-1 mouse pups, and in vivo by use of CD-1 male mice (50 and 100 mg/kg, i.p.). The results obtained showed that exposure to menthol caused dose-dependent reduction of ipsilateral and contralateral pain hypersensitivity induced by complete Freund’s adjuvant; reduction of nociceptive behavior in both phases of the formalin test; dose-dependent and Cl^−^-mediated generation of inward and outward currents in cultured dorsal horn neurons, indicating the activation of γ-aminobutyric acid type A receptors; blockage of voltage-gated sodium channels and voltage-gated calcium channels in a voltage-, state-, and use-dependent manner; decrease in the repetitive firing and action potential amplitude; reduced neuronal excitability, and interruption of spontaneous synaptic transmission of cultured superficial dorsal horn neurons. 

In the peripheral mechanisms involved in the effects displayed by menthol, activation of transient receptor potential cation channel subfamily M member 8 (TRPM8) and TRPA1 channels is believed to play an important role in menthol-induced cold hyperalgesia [97,98]. In fact, previous studies have shown that menthol reversed cinnamaldehyde-induced heat hyperalgesia, an effect that may have been a consequence of TRPA1 blockage by this compound [99,100]. In a study by Roberts and collaborators [46], the sensory effects and interactions of topically applied-TRP agonists menthol (TRPM8), capsaicin (TRPV1) and cinnamaldehyde (TRPA1) to the skin of 14 health humans were assessed through changes in thermal sensibility and contact heat-evoked potentials (CHEP). The application of menthol provoked cold hypersensitivity while cinnamaldehyde and capsaicin produced heat hyperalgesia. Furthermore, menthol and cinnamaldehyde did not exert any effect on evoked potentials, but the amplitude of CHEP and evoked pain ratings were negatively correlated after capsaicin exposure.

Other studies revealed the participation of TRPM8 as the main mediator of menthol- (or l-arginine)-induced analgesia of acute and inflammatory pain [88,101]. For instance, l-arginine (10 and 20 mg/kg, i.p.) significantly reduced pain behavior in the hot plate, acetic acid writhing and tail flick, and inflammatory (complete Freund’s adjuvant) tests. It effectively inhibited nocifensive behavior (licking, fliching or biting) caused by specific pharmacological activation of TRPV1 when administered in combination with capsaicin (5 nmoL; menthol −50 nmoL-intraplantar injection) in wild-type mice, but not in Trpm8-/-mice. Similar results were obtained following exposure of wild-type and Trpm8-/-mice to l-arginine (50 nmoL) coinjected with acrolein (25 nmoL; intraplantar), an agonist of TRPA1 in sensory nerves. More importantly, the antinociceptive activity of l-arginine was completely eliminated by genetic deletion of TRPM8, and its antinociceptive effect was restored in mice treated with AMG2850, a selective TRPM8 inhibitor. The selective activation of TRPM8 with WS-12 (a menthol derivative and a specific TRPM8 agonist) in cultured sensory neurons and in vivo also produced TRPM8-dependent antinociception of acute and inflammatory pain. l-arginine and WS-12 effects were counteracted by naloxone, indicating the participation of endogenous opioid-dependent analgesia pathways [88].

The effects of menthol on human embryonic kidney-derived 293 T cells expressing (h)TRPV1 and of capsaicin on hTRPM8 were addressed in vitro and showed that the activation of hTRPV1 currents by heat and capsaicin were suppressed by menthol, whereas activation of hTRPM8 currents were suppressed by capsaicin [101]. Moreover, an in vivo sensory irritation test carried out in Japanese males (20–30 years old; *n* = 10) demonstrated that menthol exhibited antinociceptive effect on the sensory irritation caused by a capsaicin analogue. These results suggest an interaction between TRPV1 and TRPM8 agonists and both of these channels, and since TRPM8 is not usually coexpressed with TRPV1 in primary afferent neurons [102,103], it is possible that the information transmitted by TRPM8- and TRPV1-expressing neurons could affect each other. Therefore, it is believed that menthol-induced TRPM8-mediated cold sensation could be improved by the inhibition of TRPV1 and that capsaicin-induced TRPV1-mediated heat sensation could be increased by the inhibition of TRPM8 [101]. 

### 2.6. (−)-α-Bisabolol 

(−)-α-Bisabolol (6-methyl-2-(4-methylcyclohex-3-en-1-yl)-hept-5-en-2-ol) is a sesquiterpene alcohol present in essential oils of various plant species such as *Vanillosmopsis* and *Peperonia* [104,105]. This compound has a soft floral odour, being its most popular example the species *Matriarca chamomilla*, known as chamomile [106,107]. The pharmacological properties attributed to (−)-α-bisabolol include wound healing [108], gastroprotective [109], antitumor [110], antioxidant [111], leishimanicidal [112], and peripheral nervous blocker [113].

In 2011, Leite and collaborators [114] and Rocha and collaborators [107] evidenced the antinociceptive and anti-inflammatory activities of (−)-α-bisabolol through various rodent models of nociception and inflammation. In the acetic acid-induced writhing, formalin and hot plate tests, (−)-α-bisabolol (25 or 50 mg/kg, p.o.) administered to male Swiss mice reduced the number of writhings, showing stronger effect (54.68% and 4.24%, respectively) than the reference drug indomethacin (10 mg/kg, p.o.; 48.52%); diminished paw-licking time in the second phase of the formalin test (92.95% and 92.74%, respectively), but did not affect the response time to heat in the hot plate, suggesting that (−)-α-bisabolol has no central antinocicetive effect [115]. Significant inhibition of nociceptive behavior by (−)-α-bisabolol (50, 100 or 200 mg/kg, p.o.) was also demonstrated in the cyclophosphamide and mustard oil-induced visceral nociception tests [114].

In the inflammatory models of dextran- and CG-induced paw edema, edema formation after intraplantar injection of these compounds was effectively suppressed by (−)-α-bisabolol (100 and 200 mg/kg, p.o.) pretreatment in male Swiss mice. (−)-α-Bisabolol (100 and 200 mg/kg) significantly inhibited myeloperoxidase (MPO) activity and decreased TNF-α levels in the peritoneal fluid of rats with induced peritonitis. This result indicates that (−)-α-bisabolol blocks neutrophil migration to the peritoneal cavity as indicated by MPO, a marker of the presence of these cells [115]. Moreover, the edematogenic response triggered by croton oil, arachidonic acid and phenol in the mouse ear edema was significantly decreased by topically applied (−)-α-bisabolol (0.7 and 1.4 mg/ear); however, it did not effectively reduce edema induced by caipsaicin, indicating that (−)-α-bisabolol action mechanism does not involve activation of TRPV1 receptor [114].

The antinociceptive action of (−)-α-bisabolol (50, 100 and 200 mg/kg, i.p.) was also reported by Leite and collaborators [115] in visceral nociceptive models induced by acetic acid, formalin, capsaicin and mustard oil. To investigate the mechanisms involved in its effects, prior to exposure to (−)-α-bisabolol (50 mg/kg) in the acute model of visceral nociception induced by intracolonic instillation of mustrad oil, male Swiss mice were treated with N(G)-Nitro-l-arginine methyl ester (l-NAME), yohimbine, glibenclamide, ondansetron or ruthenium red in order to confirm the participation of nitrergic, noradrenergic, K_ATP_+, 5-HT_3_, and TRPV1 receptors in the effect of this compound. Treatment with (-)-α-bisabolol and ruthenium red (non competitive antagonist of TRPV1) in combination resulted in an additive antinociceptive effect, while the results obtained with (l-NAME, NO synthase inhibitor) and ondansetron (5-HT_3_ antagonist) were inconclusive. Yohimbine (α-2 adrenoceptor) failed to antagonize (−)-α-bisabolol action, indicating that α2 adrenoceptor is not involved in the attenuation of visceral nociception. In addition, (−)-α-bisabolol (50 mg/kg) did not suppress capsaicin-induced visceral nociception, corroborating previous findings showing that this compound does not act as a TRPV1 agonist [114].

By the use of imaging, electrophysiological and biochemical methods, Nurulain and collaborators [116] showed that (−)-α-bisabolol reversibily and dose-dependently suppressed α7-nicotinic acetylcholine receptors (α7-nChRs) mediated currents in oocytes of *Xenopus laevis*. These receptors are found in the peripheral and central nervous system and are characterized by rapid desensitization and high permeability to calcium [117]. The obtained results revealed that the suppressive effect of (−-α-bisabolol (IC_50_ = 3.1µM) was not altered after injection of calcium chelator BPTA and perfusion with calcium-free solution containing barium, which is indicative of the non-involvement of endogenous calcium-dependent Cl^−^ channels in (−)-α-bisabolol activities. Furthermore, the effect of (−)-α-bisabolol on α7-nChRs were investigated in the first region in the hippocampal circuit known as CA1 region of stratum radiatum interneurons of rat hippocampal slices (whole-cell patch-clamp method) and was shown to have an inhibitory action in choline-induced currents in the CA1 interneurons [116,117].

*Stachys lavandulifolia* Vahl (Lamiaceae) is a plant used in Turkish and Iranian folk medicine as an analgesic and anti-inflammatory [118]. A study by Barreto and collaborators [118,119] provided information about the antinociceptive and anti-inflammatory effects displayed by the main compound of *S. lavandulifolia* essential oil, i.e., (−)-α-bisabolol, in models of orofacial nociception. Male Swiss mice exhibited reduced face-rubbing behavior after exposure to (−)-α-bisabolol (50 mg/kg, p.o. -first phase; 25 and 50 mg/kg, p.o. -second phase) in the formalin test. Further analysis of the pharmacological profile showed its effective inhibitory effect (25 and 50 mg/kg) on the nociceptive response in animals tested in the capsaicin- and glutamate-induced orofacial pain tests. The data presented in the pain models indicated a stronger effect of (−)-α-bisabolol in comparison with *S. lavandulifolia* essential oil. In the CG-induced pleurisy, (−)-α-bisabolol exhibited a significant anti-inflammatory activity and this result could be possibly related to the significant decrease in the level of TNF-α in pleural inflammatory exudate. No considerable alteration was observed in the level of IL-1β, but in contrast *S. lavandulifolia* essential oil markedly reduced both pro-inflammatory cytokines. These findings support the folk use of *S. lavandulifolia* and relates its antinociceptive and anti-inflammatory actions to (−)-α-bisabolol [119].

### 2.7. Cinnamaldehyde

Cinnamaldehyde (CIN) is a naturally occurring phenylpropanoid and has been described as the most important component present in the volatile oil of different cinnamon species [120]. This component contributes to the fragrance and several biological properties observed in *Cinnamomum* species, including antioxidant, antipyretic, antimicrobial and anti-inflammatory activities [121,122,123,124]. For example, *Cinnamomum zeylanicum* essential oil exhibited antinociceptive properties on acute and chronic pain in mice, and CIN seems to be involved in *C. zeylanicum* antinociceptive effect [124]. In 2011, Roberts and collaborators [46] investigated the sensory effects, with emphasis on thermal sensibility, of CIN together with capsaicin (CAP) and menthol (MEN) in a human experimental pain model. Fourteen healthy human participants received topically on the skin an unguent containing three concentrations of CIN (1%, 5% and 10%), CAP (0.075%, 1% and 3%) or MEN (2.5%, 5% and 10%). Topical CAP caused a noteworthy heat hyperalgesia. MEN provoked a cooling sensation, whereas CIN caused heat hypersensitivity at all tested concentrations. However, CIN and MEN did not present effect on evoked potentials. Further, the intensity of CIN induced heat hyperalgesia was amplified by secondary compound CAP, indicating an additive effect [46].

In another study, it was investigated the antinociceptive effect of CIN in both peripheral and central pain models (acetic acid induced writhing and Eddy’s hot plate methods, respectively) as well as its combination with two standard drugs (diclofenac sodium and pentazocine) in mice. CIN was able to reduce nociception in a dose-dependent manner, decreasing the number of writhes 54% and 81% at concentrations of 100 and 200 mg/kg, respectively. On the other hand, it was also observed a significant reduction in writhings (84.43%) when CIN (100 mg/kg) was co-administered with diclofenac sodium (2.5 mg/kg). In Eddy’s hot plate method, CIN exhibited hyperalgesic behavior when given alone as well as decreased the antinociceptive effect of pentazocine in combination group (CIN 100 mg/Kg + pentazocine 2.5 mg/Kg). These data demonstrate that CIN considerably enhanced the antinociceptive effect of diclofenac sodium at the same time as inhibited the antinociceptive action of pentazocine [125].

### 2.8. Citronellal

Citronellal (CTAL), also named rhodinal, is an acyclic monoterpenoid aldehyde known for its capacity to repel insects [126]. CTAL is one of the main responsible for the lemon-scent of many of the plants of the *Cymbopogon* genus (Poaceae), especially the species *C. nardus* [127,128], *C. winterianus* [127] and *C. citratus* [128]. In previous study, *C. winterianus* essential oil demonstrated noteworthy antinociceptive, anti-inflammatory and antioxidant properties, and the monoterpene CTAL seems to be involved in these effects [129,130]. For this reason, the antinociceptive effect of CTAL (50, 100 or 200 mg/kg, i.p.) was investigated in three experimental nociception models: formalin test, capsaicin test and glutamate-induced nociception. CTAL, in all tested doses, caused a dose-dependent reduction in the pain-related behaviors during both phases of the formalin test and was naloxone-sensitive. Similarly, this monoterpene also significantly decreased face-rubbing behavior induced by administration of capsaicin or glutamate, suggesting that CTAL possesses antinociceptive action [130].

In another study, it was examined the effect of CTAL on inflammatory nociception induced by different stimuli in mice as well as the involvement of the NO-cGMP-ATP-sensitive K^+^ channel pathway. CTAL (25, 50 or 100 mg/kg, i.p.) exhibited a significant reduction of the mechanical nociception induced by tumor necrosis factor α (TNF-α) and carrageenan in all studied doses. This monoterpene also significantly decreased the mechanical nociception in the dopamine (DA) test at doses of 25 and 100 mg/kg, and in the prostaglandin E type 2 (PGE_2_) test only at higher dose (100 mg/kg). Interestingly, pretreatment with l-NAME or glibenclamide reversed the antinociceptive effect of the CTAL (100 mg/kg) on PGE_2_-induced mechanical nociception, suggesting that CTAL inhibits mechanical nociception through the involvement of NO-cGMP-ATP-sensitive K^+^ channel pathway. Taken together, these results show the potential of CTAL for the treatment of pain [131].

### 2.9. Citronellol

Citronellol (CTOL), or dihydrogeraniol, is a natural alcoholic monoterpene found in essential oils of various aromatic plant species [132,133]. CTOL exists in nature as two enantiomers, designated *R*-(+) and *S*-(−). *R*-(+)-CTOL is widely found in citronella oils, such as *Cymbopogon winterianus*, and is the more common isomer [134]. On the other hand, *S*-(−)-CTOL is commonly found in rose [135] and geranium oils [136]. Different essential oils containing this monoterpene have been described in the literature to possess antinociceptive and anti-inflammatory effects, including *C*. *winterianus*, *C. citratus* and *Pelargonium graveolens* [130,137,138]. With this feedback, Brito and collaborators [139] evaluated the antinociceptive and anti-inflammatory activities of CTOL, in mice, using different experimental models for pain and inflammation: acetic acid-induced abdominal constrictions, formalin-induced nociception, hot plate test and carrageenan-induced pleurisy. CTOL, in all tested doses (25, 50 or 100 mg/kg, i.p), reduced the total number of writhing in acetic acid-induced abdominal constriction test. This monoterpene also decreased paw licking times during both the early and later phases of the formalin test at doses of 25, 50 and 100 mg/kg. In the hot plate test, CTOL caused a marked increase in the latency time of the animals only at the higher dose. Finally, pretreatment with CTOL was also capable to reduce, in a dose-dependent fashion, both neutrophil infiltration and the levels of TNF-α in the exudates from carrageenan-induced pleurisy. These data indicate that CTOL exhibits an interesting antinociceptive and anti-inflammatory effect, and its mechanism of action probably involves inhibition of peripheral mediators as well as central inhibitory mechanisms [139].

Giving continuity to the study performed by Brito and collaborators [139], Brito and collaborators [140] evaluated the antinociceptive effects of CTOL on orofacial nociception in mice as well as a possible central nervous system (CNS) involvement. Pretreatment with CTOL at doses of 25, 50 and 100 mg/kg (i.p.) was able to reduce nociceptive behavior in both phases of the formalin test and in the capsaicin test. Similarly, CTOL, in all assayed doses, decreased the nociceptive face-rubbing behavior in glutamate-induced orofacial nociception model. Additionally, to investigate the action of the CTOL on CNS, it was performed an immunofluorescence protocol for Fos protein. The obtained results revealed that CTOL was capable to induce a significant increase in the average number of neurons in the piriform and retrosplenial cortex, olfactory bulb and periaqueductal grey. Taken together, these data suggest that CTOL decreases orofacial nociceptive behavior and this effect involves, at least in part, the activation of CNS regions, mainly periaqueductal and grey retrosplenial cortex [140].

In another study, it was investigated the antihyperalgesic effect of CTOL in mice using several experimental models of hyperalgesia. The mechanical hyperalgesia was induced by four hyperalgesic agents: carrageenan (CG), TNF-α, PGE_2_ or dopamine. Pretreatment with CTOL, in all tested doses (25, 50 or 100 mg/kg, i.p.), was capable to attenuate mechanical hyperalgesia induced by CG, TNF-α, PGE_2_ and DA in the acute models of inflammatory nociception as well as reduce the edema formation. Additionally, it was also evaluated the involvement of the spinal cord lamina I in this antihyperalgesic effect. The immunofluorescence protocol showed that CTOL significantly decreased the average number of neurons presenting Fos protein, indicating that the action of CTOL on mechanical hyperalgesia occurs, at least in part, via inhibition of the spinal cord lamina I [141].

### 2.10. Citronellyl Acetate

Citronellyl acetate (CAT), known for its pleasant smell, belongs to the family of fatty alcohol esters and is frequently used as flavor and fragrance agent [142]. CAT is present mostly in *Eucalyptus citriodora* [143], but also is found in minor quantities in the volatile extract from dried pericarp of *Zanthoxylum schinifolium* [144]. Since there are few studies investigating the biological potential of this monoterpene and *E. citriodora* essential oil possesses antinociceptive and anti-inflammatory effects [145], Rios and collaborators [142] investigated the antinociceptive effect of CAT in both physically- and chemically-induced acute pain models as well as the possible antinociceptive mechanisms involved. CAT (25, 50, 75, 100 or 200 mg/kg, i.g.), at two higher doses, caused a significant reduction of acetic acid-induced abdominal constrictions in mice. In the formalin test, CAT (100 or 200 mg/kg, p.o.) reduced nociceptive behavior in both the early and later phases. Similarly, in the glutamate test, CAT decreased nociceptive behavior after pretreatment with the doses of 100 and 200 mg/kg (p.o.). Regarding the mechanism of action, the results showed that, at least in part, protein kinase C (PKC) and protein kinase A (PKA), transient receptor potential vanilloid 1 (TRPV1), TRPM8, acid-sensing ion (ASIC) and glutamate receptors are involved in the antinociceptive effect of CAT.

### 2.11. α-Phellandrene

α-Phellandrene (α-PHE), a cyclic monoterpene, is a natural compound that can occur in two enantiomeric forms: (−)-α-PHE and (+)-α-PHE. α-PHE is present in the volatile oil of several plants and in varied concentrations [146], such as *Anethum graveolens* (32%) [147], *Solanum erianthum* (17.5%) [148], *Schinus terebinthifolius* (15.7%) [149], *Curcuma zedoaria* (14.9%) [150], *Thymus kotschyanus* (10.8%) [151] and *Cupressus atlantica* (5.5%) [152]. Furthermore, this constituent has been associated with the antinociceptive and anti-inflammatory properties of some species, including *Matricaria chamomilla* [153], *Schinus polygamus* [154] and *Zingiber officinale* [155]. In 2012, Lima and collaborators [156] studied the antinociceptive and anti-inflammatory effects of α-PHE in five experimental nociception models (acetic acid-induced abdominal writhing, formalin test, capsaicin test, glutamate test and carrageenan-induced inflammatory hypernociception) as well as the possible antinociceptive mechanisms involved. In the writhing and capsaicin tests, α-PHE (3.125, 6.25 or 12.5 mg/kg, p.o.) exhibited a significant antinociceptive effect in all assayed doses. In the formalin test, α-PHE (50 mg/kg) reduced nociceptive behavior in both first and second phases. In the glutamate test, α-PHE (12.5 and 25 mg/kg) reduced nociceptive response in a dose dependent manner. In carrageenan-induced hyperalgesia, this monoterpene significantly reduced the inflammatory hypernociception only at dose of 50 mg/kg. In the study of the involved mechanisms, the antinociceptive effect of α-PHE was reversed by pretreatment with various drugs, such as naloxone, atropine, glibenclamide, l-arginine and yohimbine. These results indicate that α-PHE possesses noticeable antinociception and some of the possible mechanisms of action involve the opioid, nitrergic, glutamatergic, cholinergic and adrenergic systems [156].

### 2.12. α-Terpineol

α-Terpineol (α-TPN) is monoterpene alcohol that has been isolated from a variety from natural sources, such as the essential oils from *Melaleuca leucadendra* [157], *Citrus aurantium* [158] and *Nepeta dschuparensis* [159]. There are three isomers, α-, β-, and γ-TPN, the latter two differing only by the location of the double bond. Quintans-Júnior and collaborators [160] studied the antinociceptive action of α-TPN using heat-induced (hot-plate test) and chemical-induced (acetic acid, formalin, glutamate and capsaicin) nociception models in mice at different doses (25, 50 or 100 mg/kg, i.p). α-TPN, in all tested doses, exhibited a reduction of the nociceptive behavior at the early and late phases of paw licking and reduced the writhing reflex in mice (formalin and writhing tests, respectively). In the glutamate and capsaicin tests, α-TPN also reduced remarkably nociceptive response, and this inhibition of antinociceptive behavior was dose-related in capsaicin-induced nociception test. Finally, α-TPN significantly increased the latency time in the hot plate test (at only the higher dose). Taken together, these results demonstrate the potential antinociceptive properties of α-TPN [160].

### 2.13. Vanillin

Vanillin (VAN), or 4-hydroxy-3-methoxybenzaldehyde, is a phenolic aldehyde with the molecular formula C_8_H_8_O_3_. This organic compound contains three highly reactive functional groups in its structure: aldehyde, phenol and ether [161]. VAN is one of the primary chemical constituent extracted from *Vanilla planifolia* seedpods, a monocotyledonous orchid native of Central America, and is broadly employed as flavoring agents in foods, cosmetics, beverages and pharmaceuticals. Synthetic vanilla is commonly used instead of natural vanilla, since vanilla extract is so much in demand and expensive [161,162]. VAN is known to have several biological activities, including antimutagenic [163], antidepressant [162], antioxidant, hepatoprotective [164] and antitumor [165]. Further, it has been demonstrated the antinociceptive potential of VAN in acetic acid-induced visceral inflammatory pain models [166]. With this feedback, Rathnakar and collaborators [167] investigated the antinociceptive effect of VAN using Eddy’s hot plate method. Pretreatment with VAN displayed a significant increase in the latency period at both tested doses (10 or 100 mg/kg). As this experimental model is employed to evaluate the central pain, obtained results indicate a potential central antinociceptive activity probably mediated via opioid receptors [167].

In 2012, Srikanth and collaborators [168] examined the effect of VAN on acute inflammation induced by phlogistic agent carrageenan in rats. These authors observed that pretreatment with VAN (10, 100 or 200 mg/kg, p.o.) was able to significantly reduce the rat paw edema formation induced by carrageenan at 2nd, 3rd and 4th hour, and only at the higher doses (100 and 200 mg/kg). Further, there are no significant differences between the antioedematogenic effect observed at doses of 100 and 200 mg/kg [169].

In another study, it was evaluated the antinociceptive and anti-inflammatory effects of VAN on tail flick method and carrageenan-induced rat paw edema model, respectively. Pretreatment with VAN (50 or 100 mg/kg) produced a significant inhibition of pain, suggesting that antinociception in the mice tail flick test is mediated probably at the level of the spinal cord. In carrageenan-induced paw edema test, VAN caused a significant reduction in the paw volume at doses of 50 and 100mg/kg, indicating an anti-inflammatory action [169].

### 2.14. Borneol

Borneol (BOR) belongs to the family of bicyclic monoterpene alcohols and is found in the essential oil of several medicinal plants, such as *Lavandula officinalis, Matricaria chamomilla* and *Valeriana officinalis* [170,171]. There are three different isomers of BOR, d-(+)-BOR, l-(−)-BOR and isoborneol. Natural BOR contains 98% of (+)-BOR. (+)-BOR is broadly employed in food and also used in analgesic and anesthetic preparations in traditional Chinese medicine and Japanese medicine [172]. Recent studies have reported that this monoterpenoid possesses a variety of pharmacological effects, including anti-inflammatory [173], vasorrelaxant [174] and neuroprotective activities [175]. Until now, little is known about the specific role of BOR in the pharmaceutical preparations to treat painful and inflammatory conditions. With this feedback, Almeida and collaborators [175] evaluated the antinociceptive and anti-inflammatory activities of BOR, measuring nociception and inflammation in five experimental models in rodents: acetic acid-induced abdominal writhings, formalin-induced nociception, hot plate test, grip strength test and carrageenan-induced peritonitis. BOR (5, 25 or 50 mg/kg, i.p.) was able to prevent the visceral pain in acetic acid-induced abdominal writhing test in all tested doses. This monoterpene also reduced nociceptive behavior in both the early and later phases of the formalin test at doses of 5, 25 and 50 mg/kg. In the hot plate test, BOR caused a marked increase in the latency time (only at higher dose). Additionally, BOR did not cause any significant motor performance alteration in grip strength meter test. Finally, pretreatment with BOR (5, 25 or 50 mg/kg, i.p.) was able to decrease the leukocyte migration to the peritoneal cavity in peritonitis model induced by carrageenan. These findings indicate that BOR exhibits significant central and peripheral antinociceptive effects as well as anti-inflammatory activity, and without producing motor deficit [175].

In another study, it was investigated the antihyperalgesic activity of BOR on neuropathic and inflammatory pain in different animal models as well as its possible mechanisms of action. BOR (125, 250 or 500 mg/kg, p.o. or i.t.) was able to decrease mechanical hypersensitivity in both segmental spinal nerve ligation-induced neuropathic pain (SNL) and complete Freund’s adjuvant-induced chronic inflammatory pain (CFA) models and in a dose-dependent manner. Further, the antihyperalgesic action of this monoterpene, in both SNL and CFA models, was totally reversed by the convulsant alkaloid bicuculline, a selective γ-aminobutyric acid (A) receptor [GABA(A)R] antagonist. This result suggests that BOR attenuates mechanical hyperalgesia through activation spinal GABAergic transmission in the spinal cord, being a potential candidate for treating chronic pain [176]. 

### 2.15. Myrtenol

Myrtenol (MYR) belongs to the family of bicyclic monoterpene alcohols. This chiral alcohol contains two stereogenic centres and is present in the volatile oil of various aromatic species, including *Aralia cachemirica* [177] and *Tanacetum vulgare* [178]. MYR can also be obtained through the selective oxidation of the α-pinene [179], and has been reported in the scientific literature by its bioactivity [180,181,182]. Considering that this monoterpene presents important biological properties and great therapeutic potential, it was evaluated its antinociceptive and anti-inflammatory activities, in mice, using classical models of nociception (acetic acid-induced writhing, hot-plate test and paw licking induced by formalin, glutamate and capsaicin) and inflammation (paw edema induced by different agents, carrageenan-induced peritonitis, myeloperoxidase levels and cytokine measurement). Pretreatment with MYR (25–75 mg/kg, i.p.) effectively inhibited acetic acid-induced nociception; decreased time of licking the paw after the injection of the phlogistic agents glutamate, capsaicin and formalin (only in the second phase); and did not change the latency reaction time in the hot-plate test. In addition, MYR inhibited carrageenan-, histamine-, serotonin compound 48/80- and PGE_2_-induced by paw edema; and also decreased the cell counts, myeloperoxidase activity and cytokine levels of the peritoneal cavity induced by carrageenan. These results suggest that MYR attenuates the nociceptive and inflammatory responses by inhibiting cell migration and also signalling pathway of receptors involved in the transmission of pain [183].

### 2.16. Pulegone

Pulegone (PUL) is a naturally occurring organic compound present in the essential oil from several members of the mint family (Lamiaceae), such as *Minthostachys spicata* [184], *Mentha longifolia* [185] and *M. pulegium* [186]. In nature, PUL occurs in both (+)- and (−)-forms and is classified as a monoterpene ketone [4]. Further, this monoterpenoid has been recognized as being responsible for most of pharmacological effects described for species *M. longifolia* [185]. In 2011, De Sousa and collaborators [3] investigated the antinociceptive potential of PUL in chemical (formalin test) and thermal (hot plate test) models of nociception. PUL (31.3, 62.5 and 125 mg/kg, i.p.) inhibited dose-dependently both phases of the formalin test, and this effect was not blocked by opioid antagonist naloxone. In hot plate test, PUL augmented significantly the latency reaction time of mice in hot plate in all tested doses (31.3, 62.5 or 125 mg/kg), confirming that this monoterpene ketone has a central antinociceptive effect [3].

### 2.17. Citral

Citral (CIT) is a mixture of two isomers, *cis*-isomer neral and *trans*-isomer geranial, and has been described to be the most important member of the open-chain monoterpenoids. This monoterpene is found in volatile oil of several aromatic herbs, such as *Cymbopogon citratus*, a species commonly known as lemongrass [28,187]. Lemongrass tea possesses various biological properties described in literature, such as anti-inflammatory, antioxidant, anxiolytic, cytotoxic and antinociceptive activities [188]. The antinociceptive action of CIT was demonstrated in mice submitted to different experimental models of acute and chronic nociception. Pretreatment with CIT (25, 100 or 300 mg/kg, p.o.) inhibited formalin-induced licking in both the neurogenic and inflammatory phases (inhibition of 54% and 65% at 300 mg/kg, respectively); prevented and reduced mechanical hyperalgesia without producing any significant motor dysfunction, with a maximum effect at dose of 100 mg/kg; inhibited the nociceptive response (CIT 100 mg/kg) induced by glutamate (inhibition of 49%) and phorbol 12-myristate 13-acetate (PMA; inhibition of 54%); markedly attenuated the pain response (CIT 100 mg/kg) induced by *N-*methyl-d-aspartic acid (NMDA; inhibition of 54%), *trans*-1-amino-1,3-dicarboxycyclopentane (ACPD; inhibition of 77%), substance P (inhibition of 42%) or cytokine TNF-α (inhibition of 72%); and attenuated the nociception (CIT 100 mg/kg) to involve significant activation of serotonergic systems (via 5-HT_2A_ receptor). Together, these results display the potential of CIT for the treatment of inflammatory and neuropathic pain [189].

### 2.18. Thymol

Thymol (THY) is a natural monoterpene phenol derivative of cymene, and isomeric with carvacrol [190]. This monoterpene is found mainly in thyme (*Thymus vulgaris*) essential oil (approximately 47%) [191,192]. Further, THY presents various biological properties, including antinociceptive effect [191] and inhibition of inflammatory response [192]. It also known that THY inhibits nerve conduction [193], but there are no studies about how this monoterpenoid influences synaptic transmission. For this reason, Xu and collaborators [194] investigated the effect of THY on spontaneous excitatory transmission by applying the whole-cell patch-clamp technique to substantia gelatinosa (SG) neurons of adult rat spinal cord slices, aiming to comprehend how THY modulates synaptic transmission, with an emphasis on transient receptor potential (TRP) activation. It was found that THY increased the frequency of spontaneous excitatory postsynaptic current, a measure of the spontaneous release of l-glutamate onto SG neurons, by activating TRPA1 channels while producing a membrane hyperpolarization without TRP activation in SG neurons [194].

### 2.19. Limonene

Limonene (LIM) is a colorless liquid hydrocarbon belonging to the family of cyclic monoterpenes. There are two isomers, d- and l-LIM, and the more common d-isomer possesses a strong smell of orange. LIM is the major chemical component of citrus oils [195], but also is found in other aromatic plants species, including *Lippia alba* [196] and *Artemisia dracunculus* [197]. It has been reported that LIM has anti-inflammatory properties, inhibiting lipopolysaccharide (LPS)-induced production of nitric oxide, PGE_2_ and pro-inflammatory cytokines in RAW 264.7 cells [198]. For this reason, Kaimoto and collaborators [199] investigated the properties of LIM on mouse sensory neurons and heterologously expressed mouse TRP channels in vitro, as well as its nociceptive effects in vivo. The results showed that LIM directly stimulated primary sensory neurons to provoke acute pain through the activation of TRPA1 channel when was topically applied. In addition, its systemic application reduced nociceptive behaviors via H_2_O_2_-induced TRPA1 activation, and this effect is related to the inflammatory pain [199].

### 2.20. Nerol

Nerol (NER) belongs to the family of acyclic monoterpene alcohols and was originally isolated from neroli oil, hence its name. NER is found in many essential oils, such as *Agastache mexicana* [200] and *Citrus aurantium* [201]. In previous study, González-Ramírez and collaborators [202] reported the antinociceptive effect of hexane extract from *A. mexicana* aerial parts in the acetic acid-induced writhing model in rodents. As it has been reported the abundant presence of NER in this species [203], the authors suggested that this monoterpene is partially responsible by antinociceptive and anti-inflammatory activities of *A. mexicana* [202]. With this background, González-Ramírez and collaborators [203] evaluated the influence of NER in the emergence of pathological markers and hyperalgesia in oxazolone-induced colitis, as well as whether this monoterpene protects against gastric damage induced by ethanol. Pretreatment with NER (30–300 mg/kg, p.o.) significantly alleviated pathological markers (speed up body weight gain, macroscopic damage amelioration, decreased myeloperoxidase activity, reduced inflammatory parameters like disease activity index and intestinal tissue damage) observed in the oxazolone-induced colitis model. It also observed that NER (30 mg/kg) exhibited antinociceptive effect and led to a significant reduction on expression of some pro-inflammatory cytokines, like IL-13 and TNF-α. Further, NER was effective in preventing the gastric mucosa against ethanol-induced damage, starting at dose of 10 mg/kg (p.o.). These findings give evidence of the therapeutic potential of NER for the treatment of important gastrointestinal tract disorders, such as ulcerative colitis and gastric ulcers [203].

### 2.21. Anethole

Anethole (ANT), or *trans*-anethole, is an organic compound frequently used as flavoring substance. It belongs to the family of phenylpropanoids (C_6_–C_3_), a class of aromatic compounds that occurs widely in essential oils [204,205]. ANT is the main constituent of many essential oils [206], such as *Illicium verum* [207] and *Pimpinella anisum* [208], and seems to play a key role in the biological effects attributed to these oils. Previous studies have showed that ANT exhibits antioxidant [209], anti-inflammatory [210] and anesthetic [211] activities. With this feedback, Ritter and collaborators [212] examined the effects of ANT on carrageenan-induced acute inflammation and persistent inflammation induced by complete Freund’s adjuvant, two pain models of inflammatory origin. Pretreatment with ANT (125, 250, and 500 mg/kg, p.o.) provoked a noteworthy reduction of mice paw edema at doses of 250 and 500 mg/kg. Similarly, ANT also significantly decreased hypernociceptive response induced by carrageenan at doses of 250 and 500 mg/kg, but was not capable to alter the PGE_2_-induced mechanical hypernociception. Further, this phenylpropanoid was able to reduce the level of some cytokines (TNF-α, IL-1β and IL-17) at doses of 250 and 500 mg/kg, as well as inhibited the myeloperoxidase activity in all tested doses. Taken together, these findings show that ANT exhibits antioedematogenic and antihypernociceptive effects.

In another study, Ritter and collaborators [213] examined the antinociceptive activity of ANT in five experimental models of nociception: acetic acid-induced writhing, formalin test, complete Freund adjuvant-induced pain (CFA), hot-plate test and glutamate test. ANT was able to reduce the total number of writhing in the abdominal constriction model in all assayed doses (62.5, 125, 250 or 500 mg/kg, p.o.). This phenylpropanoid also decreased paw licking times during the second phase of the formalin test only at higher doses (125 and 250 mg/kg), but did not affect the nociceptive response in the first phase. Further, pretreatment with ANT significantly reduced paw edema in the glutamate test (62.5, 125 and 250 mg/kg), and decreased peripheral nociception induced by CFA (250 mg/kg). On the other hand, ANT, at different doses, did not alter the latency time in the hot plate test, confirming that ANT exhibits no central effect. These data demonstrate that ANT possesses peripheral antinociceptive action and this antinociception occurs, at least in part, by to decrease the synthesis or release of inflammatory mediators [213].

### 2.22. Nerolidol

Nerolidol (NROL), also known as peruviol, is a naturally occurring sesquiterpene alcohol present in various plants with a floral odor. NROL is the allylic isomer of farnesol (FAR) and exists in two geometric isomers, a *trans* and a *cis* form, differing only in the geometry about the central double bond [214]. This sesquiterpenoid is found in the volatile oil of many aromatic species, including *Canarium schweinfurthii* [215] and *Baccharis dracunculifolia* [216]. With this background, Fonsêca and collaborators [217] investigated the antinociceptive and anti-inflammatory activities of NROL, as well as its possible mechanisms of action, in different experimental mouse models of pain and inflammation. In the acetic acid-induced writhing test, NROL (200, 300 or 400 mg/kg, p.o.) exhibited a significant antinociceptive effect in all tested doses. In the formalin test, NROL (300 or 400 mg/kg) was able to reduce nociceptive behavior in both the first phase and the second phase. On the other hand, this sesquiterpene did not increase latency at any of the observed time points in the hot-plate test, suggesting an antinociceptive action on chemical nociception models (acetic acid-induced writhing test and formalin test), but not in the thermal nociception model (hot-plate test). Further, pretreatment with NROL decreased carrageenan-induced paw edema at doses of 200, 300 and 400 mg/kg; and inhibited the production or action of some pro-inflammatory cytokines, like TNF-α and IL-1β. Regarding the mechanism of action, the antinociceptive activity of NROL involves the GABAergic system, but not the opioidergic system or ATP-sensitive potassium (KATP) channels [217].

### 2.23. (−)-Carvone

Carvone (CAR) belongs to the family of monocyclic monoterpene ketones and exists as two optical isomers (different orientations of the isopropenyl group), d- and l-CAR. In general, these individual enantiomers possess specific biological responses, particularly toward olfactory receptors [218]. (−)-CAR is found in relevant quantities in the volatile oils from the *Mentha* genus, such as the species *M. spicata* [219,220]. This monoterpene is known to have a promising antinociceptive effect, exerting distinct effects on both central and peripheral nervous systems [221]. Until now, few studies were proposed to elucidate the potential mechanisms involved with the antinociceptive action of the (−)-CAR. For this reason, Gonçalves and collaborators [222] investigated the pharmacology of (−)-CAR in dorsal root ganglia (DRG) neurons and TRPV1-expressing HEK293 cells to verify if this compound activates TRPV1 channels. (−)-CAR did not provoke any membrane damage, presenting low cytotoxicity in both neural and epithelial cells. This monoterpene also promoted an elevation of the cytosolic calcium levels in DRG neurons through activation of TRPV1 channels. Further, activity of (−)-CAR on TRPV1 channels was examined in HEK293 cells expressing recombinant human TRPV1 channels, revealing that the increase in the calcium levels occurs in a concentration-dependent manner [222].

### 2.24. Farnesol

Farnesol (FAR) is a natural 15-carbon organic compound that is an acyclic sesquiterpene alcohol. This sesquiterpenoid is commonly found in propolis (a resinous beehive product), citrus fruits and various plant essential oils, such as *Tetradenia riparia* [223] and *Citrus* sp. [224]. In study performed by Qamar and Sultana [225], FAR showed protective efficacy against massive lung inflammation, oxidative stress and injuries induced by cigarette smoke toxicants. With this background, the antinociceptive activity of FAR was investigated in two classic behavioral models of analgesia: acetic acid-induced writhing test and the formalin-induced nociception. Pretreatment with FAR (50, 100, and 200 mg/kg, i.p.) caused a noteworthy reduction in the number of contortions in the acetic acid-induced writhing test at all assayed doses. In the formalin test, FAR was capable to inhibit both phases of the pain stimulus at doses of 100 and 200 mg/kg. These results indicate that FAR possesses antinociceptive activity and this effect was similar to that found with centrally-acting analgesic drugs, like morphine and tramadol [226].

### 2.25. β-Caryophyllene

β-Caryophyllene (β-CARY), a natural bicyclic sesquiterpene, is the main volatile constituent found in the essential oil of many common spices and food plants, such as *Cinnamomum* spp. [227], *Origanum vulgare* [228] and *Piper nigrum* [229]. In nature, is found three isomers, named (*E*)-β-CARY, (*Z*)-β-CARY (or isocaryophyllene), and α-humulene (formerly α-caryophyllene), a ring-opened isomer [230]. Further, β-CARY is known to be the main constituent of *Cannabis sativa* essential oil [231] as well as to possess antiarthritic effect [232] and noteworthy anti-inflammatory activity against carrageenan- and PGE_1_-induced edema in rats [233]. With this background, [234] examined the contribution of opioid and peripheral cannabinoid (CB) systems in the antinociceptive action produced by β-CARY as well as its action in combination with the opioid agonist morphine. β-CARY [9.0 µg/paw or 18.0 µg/paw, intraplantar (i.pl)] was able to attenuate the capsaicin-induced nociceptive behavioral response and in a dose-dependent manner. Further, β-CARY-induced antinociception was mediated by peripheral CB_2_ receptor activation, which stimulates the local release of β-endorphin, an endogenous opioid, from keratinocytes. Finally, it was also observed a synergistic antinociceptive interaction between β-CARY and morphine, fact that may be an interesting therapeutic alternative to minimize risk of undesirable side-effects caused by this opioid analgesic [234].

### 2.26. α,β-Epoxy-Carvone

α,β-Epoxy-carvone (ECAR) is a naturally occurring monocyclic monoterpene containing an epoxy group instead of the α,β-unsaturated ketone group present in CAR [235]. This monoterpene is present in the essential oils of various aromatic species such as *Kaempferia galangal* [236] and *Carum carvi* [237], but can also be obtained by organic synthesis [238]. ECAR exhibits depressor effect on CNS [180] and antimicrobial [239] and anticonvulsant activities [240]. In 2013, Da Rocha and collaborators [241] investigated the antinociceptive and anti-inflammatory effects of this monoterpenoid in four experimental mice models: acetic acid-induced writhing, formalin induced nociception, hot-plate test and peritoneal permeability induced by acetic acid. ECAR promoted a significant antinociceptive effect in the acetic acid-induced abdominal writhing test at doses of 100, 200 or 300 mg/kg (i.p.). In the formalin test, ECAR inhibited nociception in both the first phase (300 mg/kg) and second phase (200 and 300 mg/kg). In the hot-plate test, pretreatment with ECAR caused a significant latency prolongation at 30 min (100, 200 and 300 mg/kg), 60 and 120 min (300 mg/kg); and this effect was reversed by naloxone. Finally, ECAR was capable to inhibit the acetic acid-induced peritoneal capillary permeability at dose of 300 mg/kg [241].

## 3. Materials and Methods 

The compounds presented in this review were selected based on the effects shown in specific animal models for evaluation of the antinociceptive activity. Table 1 summarizes the essential oil constituents with antinociceptive activity. The search was conducted in the scientific database PubMed, focusing on works published during the last six years (January 2011 to December 2016). The data were selected using the following terms: “essential oils”, “monoterpene,” and “phenylpropanoids” refining with “analgesic” or “antinociceptive”.

## 4. Conclusions

The increasing number of studies on the antinociceptive activity of essential oil constituents shows the therapeutic potential of this chemical class. Effective in various animal models of pain and acting via different mechanisms of action, these compounds are interesting molecules for studies in clinical approaches. Despite the small amount of various antinociceptive constituents in the essential oils, it is possible, using low cost reactions, to easily synthesize some of these compounds, such as monoterpenes α, β-epoxy-carvone [180] and hydroxydihydrocarvone [242,243], which were obtained via organic synthesis. The use of these bioactive constituents as prototypes to synthesize analogous compounds is another interesting way forward in the development of new analgesic drugs. It is also necessary to investigate the toxicological aspect of essential oils. Only a few publications have shown possible toxicological effects of essential oils to humans. For example, constituents as linalool, whose antinociceptive activity in animals is well established [244,245,246,247], have been the subject of few toxicological studies in humans [248]. The standardization of the experimental protocols is also essential to establish better doses and routes of administration. In this way, it is possible to make a more appropriate comparative analysis between the oils. In addition, the investigation of the chemical composition of essential oils is important to complement the pharmacological and toxicological approach. The present review makes it possible to conclude that the structural diversity of the bioactive constituents does not allow the establishment of a chemical characteristic responsible for antinociceptive action. Advanced studies on the mechanisms of action of these constituents, together with the computational medicinal chemistry approach, may be a more efficient way to understand the chemical requirement for this pharmacological activity.

## Figures and Tables

**Table 1:** Essential oils constituents with antinociceptive activity.

Compound	Experimental Protocol	Antinociceptive Activity and/or Mechanism	Animal Tested and/or Cell Line	Reference
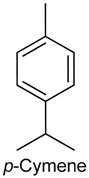	Formalin, capsaicin and glutamate-induced orofacial nociception	Decreased rubbing behavior	Male Swiss mice	[19]
	Acetic acid-induced writhing, formalin and hot plate tests CG-induced inflammation	Reduced writhes, and liking time Increased latency time of liking and jumping behavior Reduced leukocyte migration	Male Swiss mice	[20]
	Tail flick test CG, TNF-α, PGE2 and dopamine-induced hypernociception CG-induced pleurisy LPS-induced NO secretion Fos protein immunofluorescence	Increased latency time response Decreased mechanical hypernociception Reduced leukocyte and neutrophils migration and TNF-α level Reduced NO production Increased c-Fos immunoreaction	Male Swiss mice Macrophage Periaqueductal grey neurons	[25]
	Acetic acid-induced writhing and formalin tests	Reduced writhes, and liking time	Male Swiss mice	[31]
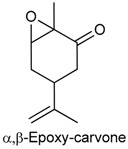	Acetic acid-induced writhing test Formalin test Hot plate test Peritoneal permeability induced by acetic acid	Reduced the number of abdominal contortions Inhibited nociception in both the first phase and second phase Caused a significant latency prolongation Inhibited the acetic acid-induced peritoneal capillary permeability	Male albino Swiss mice	[241]
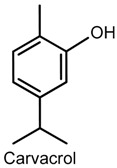	Acetic acid-induced writhing, formalin and hot plate tests	Reduced writhes, and paw-liking time Increased latency time response	Male Swiss mice	[40]
	CG, TNF-α, PGE2 and dopamine-induced hypernociception CG-induced paw edema CG-induced pleurisy LPS-induced nitrite secretion	Decreased mechanical hypernociception Decreased edema volume Reduced leukocyte migration and TNF-α level Reduced nitrite production	Male Swiss mice Macrophage	[42]
	Formalin, capsaicin and glutamate-induced orofacial nociception	Reduced face-rubbing behavior	Male Swiss mice	[43]
	Whole-cell voltage-clamp recordings	Increased secretion of l-glutamate Membrane hyperpolarization	Rat spinal cord	[44]
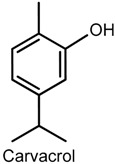	Whole-cell voltage-clamp recordings Intracellular recordings	Inhibition of excitability Generation of action potentials	Male and female Wistar rat sciatic nerve and dorsal root ganglia	[47]
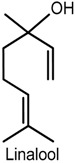	von Frey test (electronic version)	Reduced mechanical allodynia	Male ddY-strain mice (sciatic nerve ligation)	[56]
	Formalin test	Reduced licking and biting behavior	Male ddY-strain mice	[62]
	Xylene-induced ear edema and formalin-induced hind paw edema COX-2 expression and inflammatory infiltrates immunohistochemistry	Reduced edema volume Decreased COX-2 expression and inflammatory infiltrates	Male Kunming mice	[64]
	Formalin and hot plate tests c-Fos immunohistochemistry	Increased latency time of hindpaw withdrawl Increased c-Fos expression in hypothalamic orexin neurons	Wild type mice (C57BL/6) Orexin neuron-ablated mice Orexin peptide-deficient mice	[65]
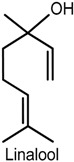	Paclitaxel-induced acute pain (von Frey filaments)	Inhibiton of mechanical allodynia and hypernociception	Male ddY-strain mice	[48]
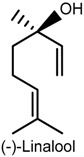	Formalin, capsaicin and glutamate-induced orofacial nociception Field potential recordings	Reduced face-rubbing behavior Inhibition of field potentials	Male Swiss mice Hippocampal dentate gyrus	[53]
	Acetic acid-induced writhing, formalin and hot plate tests CG-induced peritonitis	Reduced writhes, and paw-liking time Increased latency time response Inhibition of leukocyte migration and TNF-α level	Male Swiss mice	[67]
	Chronic noninflammatory muscle pain model c-Fos protein immunofluorescence	Inhibition of mechanical hypernociception Neuron activation	Male Swiss mice	[69]
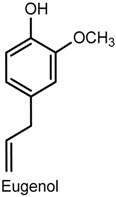	Acetic acid-induced writhing and formalin tests Substance P and glutamate-induced nociceptive behavior	Reduced writhes, and paw-liking/biting behavior Reduced nociceptive response	Male ICR mice	[73]
	Lingual irritation method	Oral irritation desensitization Increased innocuous warmth and noxious heat sensation	Human subjects	[83]
	Acetic acid-induced writhing test and glutamate-induced nociception Glutamate, AMPA, kainate, substance P and TNF-α-induced pain	Reduced writhes Reduced nociception Inhibition of biting behavior	Male Swiss mice	[71]
	Monoiodoacatate-induced osteoarthritis von Frey filaments method Spinal pain-related peptide analysis	Altered gait parameters Reduced mechanical allodynia Reduced expression of substance P and calcitonin gene-related peptide Increased dynorphin level	Sprague Dawley rats Spinal cord samples	[80]
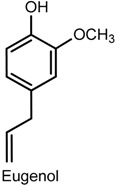	Sciatic nerve constriction von Frey and hot plate tests Biochemical assay	Inhibition of mechanical hypernociception Increased latency time response Reduced IL-1β level	Male C57BL/6J mice Sciatic nerve	[79]
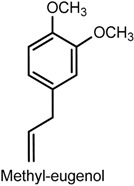	Whole-cell patch-clamp recordings	Inhibition of peripheral nerve Na_v_1.7 currents	Chinese hamster ovary cells	[86]
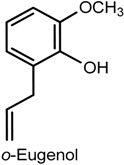	Acetic acid-induced writhing, glutamate and hot plate tests Acetic acid-induced peritoneal permeability CG-induced peritonitis	Reduced writhes and licking time Increased latency time response Inhibition of vascular permeability Reduced TNF-α and IL-1β levels	Male Swiss mice	[87]
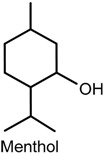	Complete Freund’s adjuvant-induced mechanical and thermal hypersensitivity Formalin-induced nociception Electrophysiological recording	Reduced mechanical and thermal allodynia Reduced paw-licking and lifting behavior Blockage of voltage-gated sodium and calcium channels Reduced neural excitability Blockage of spontaneous synaptic transmission	D1-male mice Mouse pup spinal cord dorsal horn neurons	[94]
	Quantitative sensory testing	Provoked cold hypersensitivity	Human subjects	[46]
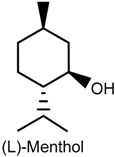	Acetic acid-induced writhing, hot plate, tail flick, and Complete Freund’s adjuvant tests Capsaicin-induced pain	Reduced writhes and licking, fliching and biting behavior	C57BL/6 wild-type mice Trp1-/- and Trpm8-/- mice	[88]
	Calcium ion-imaging Whole-cell patch-clamp recordings Sensory irritation tests	Inhibition of TRPV1 currents Reduced skin irritation	Human embryonic kidney 293 cells Human subjects	[101]
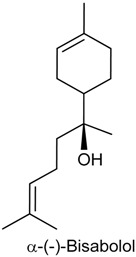	Acetic acid-induced writhing and formalin tests CG and dextran-induced paw edema CG-induced peritonitis and Neutrophil myeloperoxidade (MPO) assay	Reduced writhes and paw-licking time Inhibition of edema formation Inhibition of MPO activity Reduced neutrophil migration and TNF-α migration	Male Swiss mice Rat	[107]
	Cyclophosphamide and mustard oil-induced visceral nociception Croton oil, arachidonic acid and phenol-induced ear edema	Inhibition of nociceptive behavior Inhibition of ear edema formation	Male Swiss mice	[114]
	Acetic acid, formalin, capsaicin and mustard oil-induced visceral nocicpetion	Reduced writhes Inhibition of nociceptive behavior	Male Swiss mice	[115]
	Electrophysiological recordings Whole-cell patch-clamp recordings	Inhibition of α7-nicotinic acetylcholine receptors Inhibition of choline-induced currents	Frog oocytes (*Xenopus laevis*) Male Sprague Dawley rat hippocampus	[116]
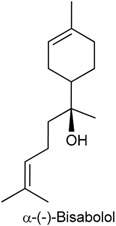	Formalin test Capsaicin and glutamate-induced orofacial nociception CG-induced pleurisy	Reduced face-rubbing behavior Inhibition of nociceptive response Reduced TNF-α level	Male Swiss mice	[119]
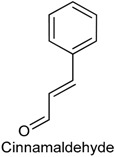	Quantitative sensory testing	Provoked heat hypersensitivity	Human subjects	[46]
	Acetic acid induced writhing Eddy’s hot plate methods	Reduced nociception Exhibited hyperalgesic behavior	Swiss albino mice	[125]
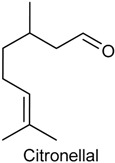	Formalin test, capsaicin test and glutamate-induced nociception	Reduced the pain-related behaviors and decreased face-rubbing behavior	Male Swiss mice	[131]
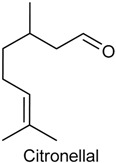	Mechanical nociception induced by CG, TNF-α, PGE_2_ and DA	Reduced mechanical nociception induced by TNF-α, PGE_2_, DA and carrageenan	Male Swiss mice	[130]
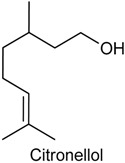	Acetic acid-induced abdominal constrictions, formalin-induced nociception, hot plate test and carrageenan-induced pleurisy	Reduced nociception, inhibited neutrophil infiltration and decreased levels of TNF-α in the exudates	Male Swiss mice	[139]
	Formaline and capsaicin tests, and glutamate-induced nociception	Decreased orofacial nociceptive behavior	Male Swiss mice	[140]
	Mechanical hyperalgesia induced by CG, TNF-α, PGE_2_ and DA	Attenuated mechanical hyperalgesia induced by CG, TNF-α, PGE_2_ and DA	Male Swiss mice	[141]
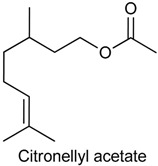	Acetic acid-induced writhing test and paw licking induced by formalin, glutamate, capsaicin, menthol, cinnamaldehyde, acidified saline, PMA and 8-Br-cAMP	Reduced the acute pain induced by acetic acid, formalin, glutamate, capsaicin, menthol, PMA and 8-Br-cAMP	Male Swiss mice	[142]
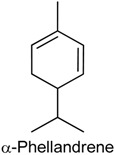	Acetic acid-induced abdominal writhing, formalin, capsaicin and glutamate tests Mechanical hypernociception and carrageenan-induced inflammatory hypernociception	Decreased the nociceptive response Reduced the hypernociception index	Male Swiss mice	[156]
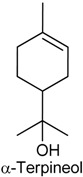	Acetic acid writhing reflex, hot plate test, and formalin-, capsaicin- and glutamate-induced nociception	Showed central analgesic properties and reduced nociceptive response induced by acetic acid, formalin, glutamate and capsaicin	Male Swiss mice	[160]
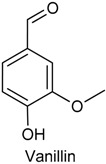	Eddy’s hot plate method	Increased the latency period, suggesting a potential central analgesic activity	Adult Wistar rats	[166]
	Carrageenan induced paw edema	Decreased the paw volume	Adult Wistar rats	[167]
	Tail flick method and carrageenan induced rat paw edema	Showed antinociceptive effect and decreased the paw volume	Adult Wistar rats	[168]
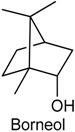	Acetic acid-induced abdominal writhings, formalin-induced nociception, hot plate test, grip strength test and carrageenan-induced peritonitis	Reduced the antinociceptive behavior, increased the latency time and decreased the leukocyte migration to the peritoneal cavity	Male Swiss mice	[175]
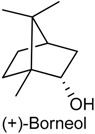	Segmental spinal nerve ligation-induced neuropathic pain and complete Freund’s adjuvant-induced inflammatory pain	Attenuated mechanical hyperalgesia through activation spinal GABAergic transmission in the spinal cord	Male adult ICR mice	[176]
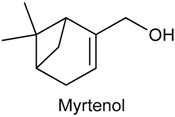	Acetic acid-induced writhing Paw licking induced by formalin, glutamate and capsaicin Hot-plate test Paw edema induced by compound 48/80, serotonin, histamine or PGE_2_ Carrageenan-induced peritonitis	Inhibited acetic acid-induced nociception Decreased time of licking the paw Did not change the latency reaction time Reduced the paw edema and Reduced the cytokine levels	Male Swiss mice	[183]
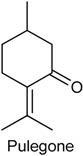	Formalin test and hot plate test	Inhibited both phases of the formalin test and augmented the latency reaction time in hot plate	Male Swiss mice	[3]
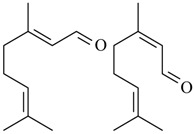 (geranial) (neral) Citral (= geranial + neral)	Acetic acid-induced writhing and formalin induced nociception	Inhibited the abdominal constrictions induced by acetic acid and reduced nociceptive behavior	Male Swiss mice and male Wistar rats	[131]
	Formalin-induced nociception, Mechanical hyperalgesia Pain behaviour induced by glutamate, capsaicin, and phorbol 12-myristate 13-acetate	Inhibited formalin-induced licking Reduced mechanical hyperalgesia Inhibited the nociceptive response	Male Swiss mice	[189]
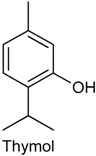	Whole-cell patch-clamp recording	Increased the frequency of spontaneous excitatory postsynaptic current	Adult Wistar rat	[194]
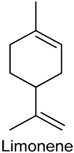	Heterologous expression in HEK293 cells H_2_O_2_-induced nociception	Stimulated primary sensory neurons Reduced nociceptive behaviors via H_2_O_2_-induced TRPA1 activation	Adult Swiss mice	[199]
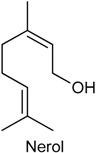	Oxazolone-induced colitis model Nociceptive behavior assay	Alleviated pathological alterations induced by oxazolone Reduced the levels of proinflammatory cytokines (IL-13 and TNF-α)	Male BALB/c mice	[203]
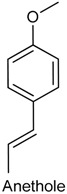	Paw edema induced by carrageenan and Complete Freund’s adjuvant Mechanical hypernociception induced by prostaglandin, carrageenan and Complete Freund’s adjuvant	Reduced the paw edema and level of some cytokines (TNF-α, IL-1β and IL-17) Decreased hypernociceptive response induced by carrageenan but was not able to alter the PGE2-induced mechanical hypernociception	Male Swiss mice	[212]
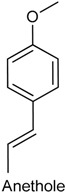	Acetic acid-induced writhing Formalin test Complete Freund adjuvant-induced pain (CFA) Hot-plate test Glutamate test	Reduced the total number of writhing Decreased paw licking times during the second phase of the formalin test Decreased peripheral nociception induced by CFA Did not alter the latency time in the hot plate test Reduced paw edema in the glutamate test	Male Swiss mice	[213]
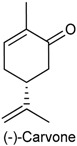	Dorsal root ganglia (DRG) neurons and TRPV1-expressing HEK293 cells	Did not provoke any membrane damage and promoted an elevation of the cytosolic calcium levels in DRG neurons	Old Wistar rat	[222]
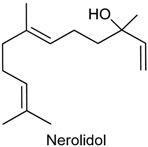	Acetic acid-induced writhing test ormalin test Hot plate test Carrageenan-induced paw edema Carrageenan-induced peritonitis	Decreased the total number of writhing Reduced the licking time in both phases Did not increase latency in the hot-plate test Decreased carrageenan-induced paw edema Inhibited the production or action of some proinflammatory cytokines	Male Swiss mice	[217]
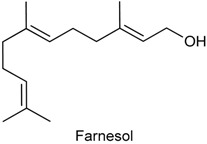	Acetic acid-induced writhing test Formalin-induced nociception	Reduced the number of abdominal contortions Inhibited both phases of the pain stimulus	Male albino Swiss mice	[226]
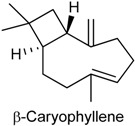	Capsaicin test	Attenuated the capsaicin-induced nociceptive response and this effect was mediated by peripheral CB_2_ receptor activation	Male Swiss mice	[234]

CG: carrageenan; TNF-α: tumor necrosis factor α; PGE_2_: prostaglandin E type 2; LPS: lipopolysaccharide; NO: nitric oxide; COX-2: ciclo oxigenase type 2; ICR: Institute of Cancer Research; AMPA: adenosine 3′,5′-cyclic monophosphate; IL: interleucin; TRPV: transient receptor potential vanilloid; DA: dopamine; PMA: phorbol myristate acetate; 8-Br-cAMP: 8-bromo-adenosine 3′,5′-cyclic monophosphate; GABAergic: γ-aminobutyric acid; TRPA: transient receptor potential ankyrin; CB_2_: cannabinoid receptor type 2.
